# Coronavirus-Induced Cardiac Tamponade in a Healthy 29-Year-Old Patient

**DOI:** 10.7759/cureus.63508

**Published:** 2024-06-30

**Authors:** Beth Schwartz, Waleed Rehman, Ramtej Atluri, Kaitlin Natole, Miriam Levine

**Affiliations:** 1 Internal Medicine, Ascension St. John Hospital, Detroit, USA; 2 Cardiology, Ascension St. John Hospital, Detroit, USA; 3 Emergency Medicine, Ascension St. John Hospital, Detroit, USA; 4 Infectious Disease, Ascension St. John Hospital, Detroit, USA

**Keywords:** severe acute respiratory syndrome coronavirus 2, cardiovascular intensive care unit, viral pericarditis, covid 19, cardiac tamponade

## Abstract

Pericarditis and pericardial effusion related to COVID-19 can lead to cardiac tamponade. Most case reports describe these complications in middle-aged or elderly patients. This case highlights a 29-year-old healthy patient who developed cardiac tamponade requiring an emergent pericardial window within one week of COVID-19 infection. This case also highlights the utility of point-of-care ultrasound in diagnosing serious COVID-19 complications.

## Introduction

COVID-19 infection is responsible for over six million hospitalizations in the United States at the time this manuscript was written [1]. Cardiac complications, including pericarditis and pericardial effusion, have been identified in many patients afflicted with the virus, with some studies estimating up to 14% of patients may develop pericardial effusions [2].

A variety of case reports have been written detailing pericardial effusion and tamponade in patients with COVID-19; however, the vast majority highlight patients who are at least 40 years old. Pericardial effusions and pericarditis have been identified in both acute COVID-19 infections and as long-term complications of the viral illness. We present a case of COVID-19-related cardiac tamponade in a healthy 29-year-old female. This patient initially had mild symptoms; however, they became acutely dyspneic and experienced syncopal episodes. Our case highlights the importance of a multidisciplinary approach to COVID-19 infection, and the potential mechanisms of COVID-19-related pericarditis and explores the utility of point-of-care ultrasound (POCUS) in cases of pericarditis and tamponade.

## Case presentation

**Figure 1:**
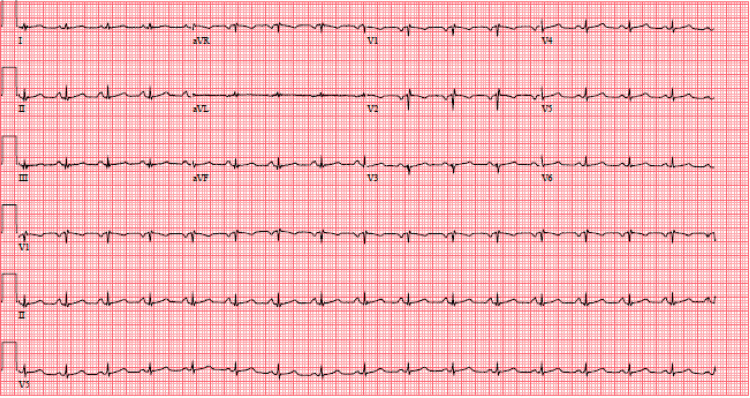
Electrocardiogram displaying normal sinus rhythm with low-voltage QRS complexes.

A 29-year-old female with no reported past medical history was diagnosed with COVID-19 approximately one week before presentation. She was feeling slightly fatigued and was managing her symptoms at home. A few days later, she began to feel increasingly short of breath. She presented to the emergency department where she had two episodes of syncope. She endorsed palpitations, shortness of breath, and diffuse chest tightness. She denied orthopnea; however, she stated she was more comfortable in the tripod position. On arrival to the emergency department, vital signs were as follows: blood pressure 97/79 mmHg, heart rate 102 beats per minute, afebrile, and oxygen saturation 100% on a 15-L high-flow nasal cannula. She appeared diaphoretic with dry mucous membranes. A cardiac exam was significant for tachycardia; however, no pericardial rub was auscultated. Laboratory results were significant for a high-sensitivity troponin of 961 ng/L, lactic acid of 3.9 mmol/L, and white blood cell count of 18.20 K/mcL (Table 1). The electrocardiogram displayed low-voltage complexes with a ventricular rate of 97 beats per minute with possible left atrial enlargement (Figure 1).

**Table 1:** Laboratory values.

Laboratory test	Result	Reference range
Hemoglobin (g/dL)	14.5	12.0-16.0
White blood cell count (K/mcL)	18.20	4.0-11.0
Platelet count (K/mcL)	197	150-400
Creatinine (mg/dL)	0.90	0.70-1.20
Sodium (mmol/L)	140	135-145
Potassium (mmol/L)	4.1	3.5-5.0
Bicarbonate (mmol/L)	20	23-34
Anion gap (mmol/L)	13	4-14
Magnesium (mEq/L)	1.6	1.3-1.9
Lactic acid (mmol/L)	3.9	<2.0
High-sensitivity troponin (ng/L)	961	<14
C-reactive protein (mg/L)	16.4	0-10
Antinuclear antibody	Negative	Negative

**Figure 2:**
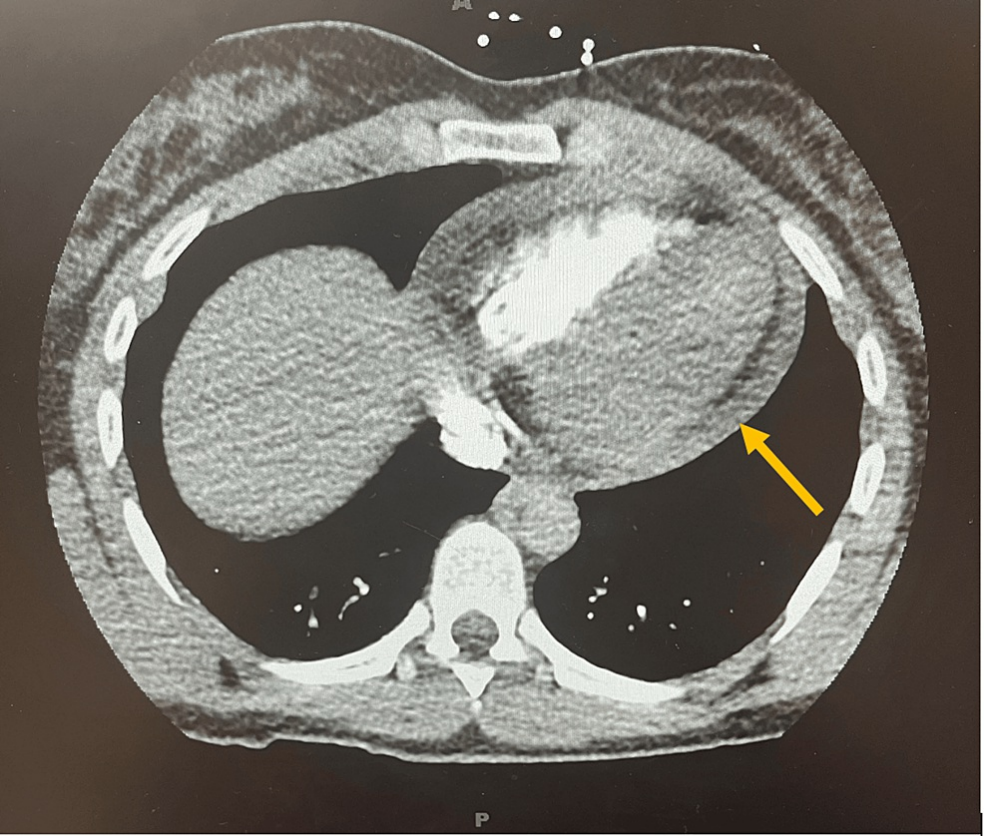
Chest computed tomography with angiography (CTA) showing a large pericardial effusion (yellow arrow).

Computed tomography angiography (CTA) was obtained due to concern for pulmonary embolism (PE). The study was negative for PE; however, it showed a large pericardial effusion (Figure 2). Bedside POCUS showed a large pericardial effusion with concern for tamponade physiology (Videos 1-2). This was evidenced by collapse of the right ventricle. Cardiothoracic surgery performed an emergent pericardial window with drainage of 150 mL of serous fluid. Cytology was negative for malignant cells. She improved over several days, was able to transition from high-flow oxygen to room air, and was discharged home with colchicine 0.6 mg twice daily.

**Video 1:** Point-of-care ultrasound showing a parasternal long view revealing a large pericardial effusion with RV collapse, as well as a left-sided pleural effusion. RV, right ventricular

**Video 2:** Point-of-care ultrasound showing an apical four-chamber view with collapse of the RV. RV, right ventricle

## Discussion

To date, there have been over six million hospitalizations nationwide for COVID-19-related illnesses [1]. COVID-19 affects a variety of organ systems, including the cardiovascular system. Pericarditis and subsequent pericardial effusion have been identified in both acutely infected patients with COVID-19 and those who were weeks into their illness [3]. A variety of mechanisms have been proposed, including binding of the virus to angiotensin-converting enzyme 2 (ACE2) receptors and activation of a signal cascade leading to myocardial injury, which facilitates effusion development. Other theories involve the cytokine storm phenomenon wherein the release of chemical messengers such as tumor necrosis factor-alpha (TNF-a), interleukin-1 (IL-1), IL-6, and IL-8 leads to inflammation. In these scenarios, the effusion is hypothesized to be a result of pericardial inflammation [3].

The diagnosis of pericarditis requires at least two out of four criteria be fulfilled: chest pain, significant new or worsening pericardial effusion, pericardial rub, or saddle-shaped ST elevation or PR depression on electrocardiogram [4]. Our patient had chest pain and a new pericardial effusion, fulfilling two out of four criteria. Cardiac tamponade is caused by accumulation of fluid in the pericardial space, leading to compression of the heart [5]. This was evidenced in our patient by bedside POCUS showing a pericardial effusion with collapse of the right ventricle (Videos 1-2). Although nonspecific, collapse of the right atrium and ventricle are among the most characteristic signs of tamponade [5]. POCUS, in this case, proved invaluable as this patient presented in the middle of the night without an echocardiography technician available. The American Society of Echocardiography has identified several indications for cardiac POCUS in acute COVID-19 infections, including identification of pericardial effusion or myocarditis [6].

In one study analyzing 340 patients with pericarditis, 12.5% of cases were attributed to inflammatory causes, including viral infection [7]. A retrospective study of 530 patients with COVID-19 infection found that pericarditis and pericardial effusion were present in 3.2% and 14% of patients, respectively [2]. Mortality was found to be higher among the group with pericardial effusions, although tamponade was not the cause of death in any of these patients [2].

This case describes a previously healthy young patient diagnosed with COVID-19 who developed tamponade within one week of diagnosis. Most case reports on this condition are in patients who are at least in the middle-age demographic. A systematic review of 30 cases describing tamponade secondary to COVID-19 infection found only three cases in patients less than 40 years old, one of whom was a 30-year-old with type 1 diabetes mellitus [3,8]. Two young female patients without comorbidities, 29 and 30 years old, were identified in this review. The 29-year-old patient was not diagnosed until three weeks after initially testing positive [9]. The 30-year-old patient described in the case written by Walker et al. is most similar to our patient. Both were young, previously healthy females who developed pericarditis, pericardial effusion, and tamponade within one week of diagnosis [10].

## Conclusions

A high index of suspicion is necessary to diagnose tamponade, especially in young patients without cardiac risk factors. Further research is needed to investigate what mechanisms may predispose young, healthy patients to serious complications of COVID-19, including cardiac tamponade. Additionally, studies to identify which risk factors may lead patients to develop complications such as acute or sub-acute pericarditis could enable providers to risk-stratify patients upon diagnosis. Finally, there are limited data on cardiac POCUS as it relates to COVID-19 outcomes. Subsequent research into these questions will be useful as the healthcare community continues to improve COVID-19 management.
